# Modulating Endoplasmic Reticulum Chaperones and Mutant Protein Degradation in GABRG2(Q390X) Associated with Genetic Epilepsy with Febrile Seizures Plus and Dravet Syndrome

**DOI:** 10.3390/ijms25094601

**Published:** 2024-04-23

**Authors:** Sarah Poliquin, Gerald Nwosu, Karishma Randhave, Wangzhen Shen, Carson Flamm, Jing-Qiong Kang

**Affiliations:** 1 Neuroscience Graduate Program, Vanderbilt University, Nashville, TN 37232, USA; sarah@combinedbrain.org; 2 Vanderbilt Brain Institute, Vanderbilt University, Nashville, TN 37232, USA; gnwosu18@email.mmc.edu; 3 Department of Neuroscience and Pharmacology, Meharry Medical College, Nashville, TN 37208, USA; 4 Department of Neurology, Vanderbilt University Medical Center, 465 21st Ave South, Nashville, TN 37232, USA; karishma.randhave@vumc.org (K.R.); wangzhen.shen@vumc.org (W.S.); carson.w.flamm@vanderbilt.edu (C.F.); 5 Department of Pharmacology, Vanderbilt University, Nashville, TN 37232, USA; 6 Vanderbilt Kennedy Center of Human Development, Vanderbilt University, Nashville, TN 37232, USA

**Keywords:** epilepsy, GABA_A_ receptor, endoplasmic-reticulum-associated protein degradation (ERAD), E3 ubiquitin ligase, proteostasis, intracellular trafficking, Dravet syndrome

## Abstract

A significant number of patients with genetic epilepsy do not obtain seizure freedom, despite developments in new antiseizure drugs, suggesting a need for novel therapeutic approaches. Many genetic epilepsies are associated with misfolded mutant proteins, including *GABRG2(Q390X)*-associated Dravet syndrome, which we have previously shown to result in intracellular accumulation of mutant GABA_A_ receptor γ2(Q390X) subunit protein. Thus, a potentially promising therapeutic approach is modulation of proteostasis, such as increasing endoplasmic reticulum (ER)-associated degradation (ERAD). To that end, we have here identified an ERAD-associated E3 ubiquitin ligase, HRD1, among other ubiquitin ligases, as a strong modulator of wildtype and mutant γ2 subunit expression. Overexpressing HRD1 or knockdown of HRD1 dose-dependently reduced the γ2(Q390X) subunit. Additionally, we show that zonisamide (ZNS)—an antiseizure drug reported to upregulate HRD1—reduces seizures in the *Gabrg2^+/Q390X^* mouse. We propose that a possible mechanism for this effect is a partial rescue of surface trafficking of GABA_A_ receptors, which are otherwise sequestered in the ER due to the dominant-negative effect of the γ2(Q390X) subunit. Furthermore, this partial rescue was not due to changes in ER chaperones BiP and calnexin, as total expression of these chaperones was unchanged in γ2(Q390X) models. Our results here suggest that leveraging the endogenous ERAD pathway may present a potential method to degrade neurotoxic mutant proteins like the γ2(Q390X) subunit. We also demonstrate a pharmacological means of regulating proteostasis, as ZNS alters protein trafficking, providing further support for the use of proteostasis regulators for the treatment of genetic epilepsies.

## 1. Introduction

Genetic epilepsies (GE) are associated with mutations in genes encoding a variety of proteins, and these mutations can impact protein folding, trafficking, and stability. Many of these epilepsy-associated mutations affect the main inhibitory pathway in the central nervous system, the GABAergic neurotransmission system. The GABA type A receptor (GABA_A_R) is the primary receptor mediating GABAergic signaling and is typically composed of two α subunits, two β subunits, and one γ subunit. A number of mutations have been identified in the γ2 subunit-encoding gene *GABRG2*, and these mutations are associated with a range of neurological phenotypes, from anxiety and childhood absence epilepsy on one end to Dravet syndrome on the other end [1,2,3,4,5,6,7,8,9,10,11]. While some of these identified mutations are missense mutations substituting a single amino acid, several nonsense mutations have also been reported [12,13,14,15]. Nonsense mutations in *GABRG2* are of particular scientific interest as they not only lead to loss of functionality of the shortened γ2 protein but can also alter the trafficking and degradation of the partnering α and β subunits that together comprise the whole GABA_A_R [16,17,18,19,20]. One such mutation, *GABRG2(Q390X)*, is associated with genetic epilepsy febrile seizure plus (GEFS+) and Dravet syndrome, a severe developmental and epileptic encephalopathy (DEE) [15,21], and the resulting γ2(Q390X) subunit dominant-negatively suppresses the wildtype GABA_A_ receptors and disturbs proteostasis in the endoplasmic reticulum (ER) [22].

The γ2(Q390X) subunit is misfolded, as the truncation deletes the 78 amino acids constituting the majority of the intracellular loop and all of the fourth transmembrane domain, dramatically altering the conformation of the remaining polypeptide [15,16,21]. However, the γ2(Q390X) subunit is still capable of interacting with the α1 and β2 subunits, as well as the wildtype γ2 subunit [16,18]. This thus results in a dominant-negative suppression of the biogenesis and trafficking of the wildtype GABA_A_R, leading to fewer receptors and a more severe disease phenotype compared to simple haploinsufficiency conditions in heterozygous knockout *Gabrg2^+/−^* mice [21]. In addition to impaired GABA_A_R function, a chronic presence of misfolded proteins in the ER can cause ER stress, and sustained ER stress can result in apoptosis [23,24,25]. Neuronal death is indeed seen in *Gabrg2^+/Q390X^* [21]. Thus, the removal of the mutant protein could be beneficial for the remaining receptor channel function and disease outcome.

In this study, we have investigated a potential method of promoting the degradation of the γ2(Q390X) subunit, utilizing the endogenous ER-associated degradation (ERAD) mechanism. Membrane proteins, such as the GABA_A_R subunits, are folded in the ER before passing through the rest of the secretory pathway [26,27,28,29]. Protein quality control mechanisms target terminally misfolded proteins for degradation [28,30]. A key step in this process is the ubiquitination of the misfolded protein by an E3 ubiquitin ligase [30,31,32]. E3 ligases are known to be involved in many diverse neurological disorders, including Parkinson’s disease, Alzheimer’s disease, Angelman syndrome, Fragile X syndrome, and genetic epilepsies [31,32,33,34]. Here, we probed the ability of several E3 ligases to alter expression of the γ2(Q390X) mutant subunit and identified HRD1 as the most efficient in the disposal of the mutant γ2 subunit. We present evidence that a drug previously reported to upregulate HRD1 reduces seizures in *Gabrg2^+/Q390X^* mice and facilitates surface trafficking of GABA_A_R subunits.

## 2. Results

### 2.1. The GABRG2(Q390X) Mutation Results in γ2 Dimers and Reduces Expression of the Partnering α1 and β2 Subunits

**Figure 1:**
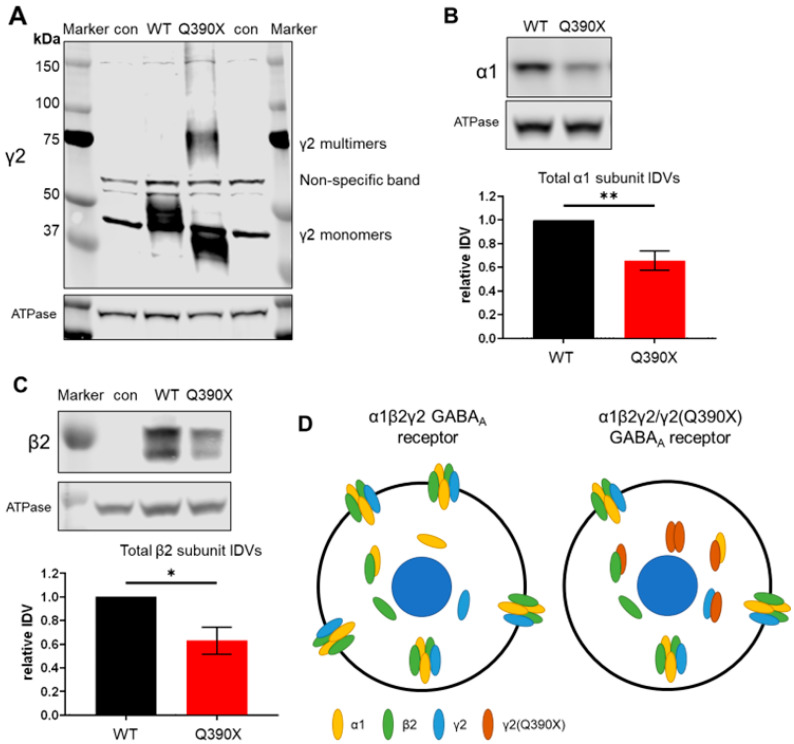
The GABRG2(Q390X) mutation resulted in the γ2 subunit dimers and reduced expression of α1 and β2 subunits. A-C. HEK293T cells were transfected with wildtype γ2 or γ2 truncation mutations and wildtype α1 and β2 (total cDNA: 3 µg per 60 mm dish). Con is untransfected control. 48 h after transfection, cells were harvested and lysed. Lysates were subjected to SDS-PAGE. (**A**) Immunoblot for γ2 (1:1000). Monomers of γ2 can be seen on the bottom half of the membrane, and dimers and larger multimers can be seen on the top half. γ2 runs slightly below the predicted size of 55 kDa, consistently running at ~45 kDa. The band near 60 kDa is nonspecific. (**B**) Immunoblot for α1 (1:500) and graph showing integrated density values (IDVs) normalized to the loading control and then to the wildtype α1β2γ2 condition. (**C**) Immunoblot for β2 (1:1000) and graph of normalized IDVs. (**D**) Cartoon demonstrating that the γ2(Q390X) mutant subunit forms oligomers and retains partnering wildtype α1, β2, and γ2 subunits intracellularly, preventing proper trafficking of GABA_A_Rs to the cell surface. N = 5–6 separate transfections. Unpaired *t*-tests were used to evaluate statistical significance. * *p* < 0.05, ** *p* < 0.01. Values are expressed as the mean ± S.E.M.

We have demonstrated that the mutant γ2(Q390X) subunit is prone to self-dimerization, resulting in dimers and larger oligomers (Figure 1A) [16,21,35]. Because this protein cannot fold properly, it is retained in the ER, which also results in the ER retention of partnering α1 and β2 subunits [16,18]. Due to the trafficking impediment, the α1 and β2 subunits are subjected to increased degradation, lowering the total protein expression of these subunits [16,36]. In line with these previous findings, HEK293T cells expressing α1β2γ2(Q390X) GABA_A_R had a reduction in the expression of the α1 subunit (WT vs. Q390X: 1 vs. 34.5% ± 8.1%, *p* = 0.0017) and β2 subunit expression (WT vs. Q390X: 1 vs. 36.9 ± 11.6%; *p* = 0.0128), compared to cells expressing α1β2γ2 GABA_A_R (Figure 1B,C). This suggests that the presence of the mutant γ2(Q390X) subunit suppressed the biogenesis and, consequently, the function of the partnering subunits like the α1 and β2 or γ2 subunits (Figure 1D).

### 2.2. Overexpression of an E3 Ubiquitin Ligase Increased γ2(Q390X) Subunit Degradation

Misfolded proteins are tagged for degradation by the addition of ubiquitin proteins, which are added by substrate-specific E3 ubiquitin ligases [30]. Although hundreds of E3 ligases have been identified, their substrates are not always known. Therefore, we tested several E3 ligases that are known or suspected to ubiquitinate proteins related to the γ2 subunit: HMG-CoA reductase degradation protein 1 (HRD1), ubiquitin-protein ligase E3A (UBE3A), neural precursor cell-expressed developmentally downregulated gene 4-like (NEDD4L, also called NEDD4-2), and ring finger protein 34 (RNF34).

HRD1, also called synoviolin 1 (SVN1), is an ERAD-associated E3 ubiquitin ligase known to ubiquitinate the α1 subunit, which has high similarity to the γ2 subunit [26,37]. UBE3A, also known as E6-associated protein (E6AP), is postulated to influence *GABRB3* expression via repressor element 1 (RE1)-silencing transcription factor (REST) [38]. Additionally, UBE3A interacts with plic-1, which is known to stabilize the β3 subunit [39,40]. Indeed, unpublished data from our previous study of the *Ube3a^−/−^* mouse [41] suggested that Ube3a regulates expression of the β3 subunit in mice. NEDD4L, meanwhile, is an epilepsy-associated protein that regulates expression of the α-amino-3-hydroxy-5-methyl-4-isoxazolepropionic acid (AMPA) receptor subunit GluA1 [42,43,44]. GluA1, also referred to as GluR-1, has a 20% sequence identity to the γ2 GABA_A_R subunit and several similar domains. Additionally, NEDD4L regulates neuron excitability independently of AMPA receptors, which opens the possibility of other neurotransmitter receptors such as GABA_A_R also interacting with NEDD4L [45]. Thus, we chose to investigate whether HRD1, NEDD4L, or UBE3A overexpression affects the expression of the γ2(Q390X) mutant subunit. Finally, RNF34 has been shown to ubiquitinate the γ2 subunit for degradation, but RNF34 specifically interacts with a portion of the C-terminal region that is missing in the γ2(Q390X) subunit (amino acids 362-404) [46]. We thus included RNF34 to confirm if it is indeed not capable of decreasing the expression of the γ2(Q390X) subunit.

**Figure 2:**
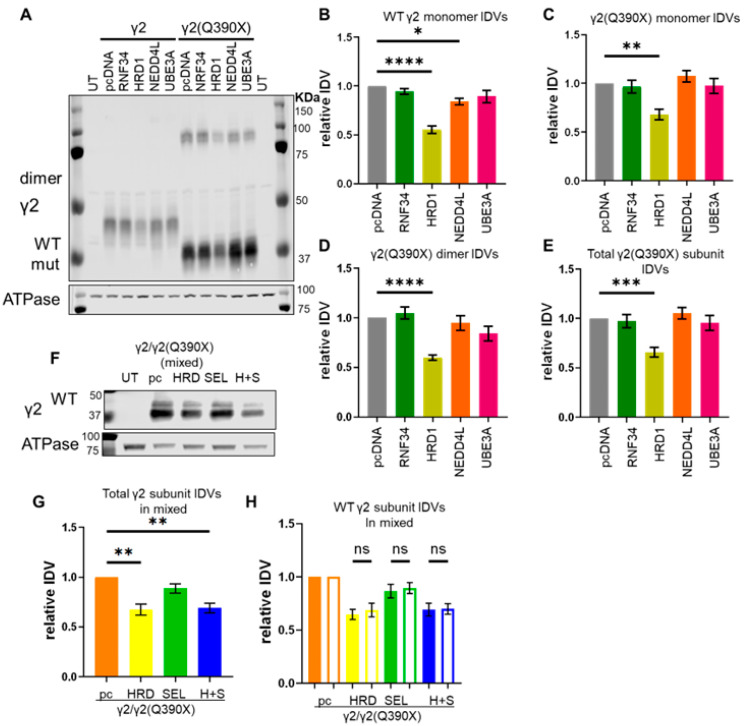
Overexpression of the E3 ubiquitin ligase HRD1-enhanced γ2(Q390X) subunit degradation. (**A**–**E**) HEK293T cells were transfected with γ2 or γ2(Q390X) (3 μg) and pcDNA, HA-RNF34, HRD1-myc, HA-NEDD4L, or UBE3A-HA (0.5 μg). (**A**) Here, 48 h post-transfection, cells were collected, lysed, and subjected to SDS-PAGE. The membrane was immunoblotted with an anti-γ2 antibody (1:1000). (**B**) A graph of the IDVs of the wildtype γ2 band ~45 kDa, normalized first to the loading control ATPase (1:1000) and then to the γ2 + pcDNA condition. (**C**) A graph of the IDVs of the γ2(Q390X) monomers (lower band, 39 kDa). (**D**) A graph of the IDVs of the γ2(Q390X) dimers (upper band, ~80 kDa). E. A graph of the total γ2(Q390X) signal, the sum of the upper and lower bands. (**C**–**E**) IDVs were normalized to ATPase (1:1000) and then to the γ2(Q390X) + pcDNA control condition. (**F**–**H**) HEK293T cells were transfected with γ2 and γ2(Q390X) (1.5 μg each). They were cotransfected with HRD1-myc (labeled HRD) or HA-SEL1L (labeled SEL) or both HRD1-myc and HA-SEL1L (labeled H+S), using 0.5 μg of these plasmids. Total cDNA was normalized to 4 μg with empty vector pcDNA. pc is γ2 and γ2(Q390X) + pcDNA. UT are untransfected controls. (**F**) An SDS-PAGE membrane was immunoblotted with γ2 (1:1000). Wildtype γ2 runs at ~45 kDa, and γ2(Q390X) runs at ~39 kDa and can thus be seen separately. (**G**) Graph showing γ2 IDVs normalized first to the loading control, ATPase (1:1000), and then to the control condition, γ2 + γ2(Q390X) + pcDNA. The sum of both wildtype γ2 and mutant γ2(Q390X) is presented here. (**H**) Normalized IDVs of the wildtype γ2 and mutant γ2(Q390X) bands in (**F**) are shown individually, with wildtype as the solid bars and mutant as the outlined bars. N = 8 separate transfections for (**A**–**E**); 7 separate transfections for (**F**–**G**). One-way ANOVA and Šídák’s (**A**–**E**) or Tukey’s (**F**–**G**) test for post-hoc analysis, corrected for multiple comparisons, were used to evaluate statistical significance. * *p* < 0.05, ** *p* < 0.01, *** *p* < 0.001, **** *p* < 0.0001. Values are expressed as the mean ± S.E.M.

Each of these E3 ligases was co-transfected with the wildtype γ2 or γ2(Q390X) subunits, and the expression of the γ2 protein was assessed by Western blot. Both HRD1 and NEDD4L overexpression decreased the amount of γ2 protein, although HRD1 was more effective than NEDD4L at reducing γ2 subunit expression (compared to pcDNA control 1 ± 0: RNF34 0.943 ± 0.030, *p* = 0.770; HRD1 0.552 ± 0.038, *p* < 0.0001; NEDD4L 0.842 ± 0.034, *p* = 0.026; UBE3A 0.894 ± 0.063, *p* = 0.219) (Figure 2A,B). Likewise, HRD1 overexpression reduced the γ2(Q390X) subunit expression, while other E3 ligases had no effect on the γ2(Q390X) subunit. Because the γ2(Q390X) subunit is often detected as dimers, we quantified monomers, dimes, and the total amount of γ2(Q390X) protein. For γ2(Q390X) subunit monomers, compared to pcDNA control 1 ± 0: RNF34 0.967 ± 0.067, *p* = 0.991; HRD1 0.681 ± 0.056, *p* = 0.0017; NEDD4L 1.074 ± 0.058, *p* = 0.849; UBE3A 0.975 ± 0.076, *p* = 0.997 (Figure 2C). Similarly, for γ2(Q390X) dimers, compared to pcDNA control 1 ± 0: RNF34 1.047 ± 0.060, *p* = 0.958; HRD1 0.598 ± 0.027, *p* < 0.0001; NEDD4L 0.948 ± 0.075, *p* = 0.940; UBE3A 0.842 ± 0.072, *p* = 0.182 (Figure 2D). And, for total γ2(Q390X) expression, compared to pcDNA control 1 ± 0: RNF34 0.974 ± 0.068, *p* = 0.996; HRD1 0.657 ± 0.050, *p* = 0.0005; NEDD4L 1.053 ± 0.058, *p* = 0.943; UBE3A 0.957 ± 0.075, *p* = 0.972 (Figure 2E). Expression of each E3 ligase was confirmed with the corresponding antibody (Supplementary Figure S1).

It has been reported that suppressor of lin-12-like protein 1 (SEL1L) stabilizes HRD1 [47]. We thus conjectured that overexpression of both HRD1 and SEL1L will have an additive effect on the γ2(Q390X) subunit degradation. Because patients with *GABRG2* mutations are heterozygous for their mutations, we mimicked this heterozygosity by transfecting HEK293T cells with equal amounts of wildtype and mutant γ2 subunit cDNA (1.5 μg each). Cells were cotransfected with 0.5 μg of HRD1-myc, HA-SEL1L, or both HRD1-myc and HA-SEL1L, and vector pcDNA was used to normalize the transfection to 4 μg for all conditions. Expression of total γ2 protein—wildtype and mutant combined—was found to be decreased in cells transfected with HRD1-myc (0.675 ± 0.055, *p* = 0.0001) or HRD1-myc + HA-SEL1L (0.690 ± 0.048, *p* = 0.0002), but not HA-SEL1L alone (0.889 ± 0.046, *p* = 0.284) (Figure 2F,G, Supplementary Figure S2). It is of note that the wildtype γ2 and γ2(Q390X) subunits were affected similarly: HRD1-myc (0.646 ± 0.046 WT vs. 0.688 ± 0.065 Q390X, *p* = 0.906) and HRD1-myc + HA-SEL1L (0.692 ± 0.059 WT vs. 0.698 ± 0.050 Q390X, *p* = 0.999) reduced the levels of wildtype and mutant protein equally, and transfection with HA-SEL1L had no effect on either form of γ2 subunit (0.865 ± 0.065 WT vs. 0.893 ± 0.050 Q390X, *p* = 0.971) (Figure 2H). This is likely because both the wildtype and the mutant γ2 subunits are retained inside the ER when expressed alone.

### 2.3. A Suggested HRD1 Upregulation, ZNS, Increased Surface Expression of GABA_A_R Subunits In Vitro

ZNS is an antiseizure drug and has been reported to increase HRD1 levels [48,49,50,51]. Therefore, we predicted that ZNS-induced upregulation of HRD1 would have similar effects on GABA_A_R subunits as HRD1 overexpression. Based on effective doses used in other independent studies, a dose of 3 μM was chosen for these experiments [49,51,52], as we identify it as the most optimal.

HEK293T cells were transfected with the wildtype α1β2γ2 receptors or the mutant α1β2γ2/γ2(Q390X) and treated with ZNS (3 μM, 24 h) or cotransfected with HRD1-myc, HA-SEL1L, or both HRD1-myc and HA-SEL1L. These conditions were compared to wildtype α1β2γ2. As before, empty vector pcDNA was used to normalize the total cDNA amount used for transfection.

**Figure 3:**
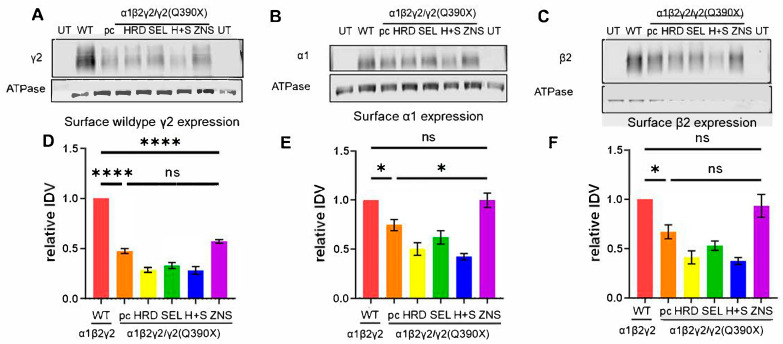
ZNS facilitated the trafficking of GABAAR subunits to the cell surface. (**A**–**C**) HEK293T cells were transfected with α1, β2, and γ2 subunit cDNAs and empty vector pcDNA (3 μg each) (labeled as WT). UT are untransfected controls. For all other conditions, the cells were transfected with α1, β2, γ2, and γ2(Q390X) (3:3:1.5:1.5 μg). They were cotransfected with HRD1-myc (labeled HRD) or HA-SEL1L (labeled SEL) or both HRD1-myc and HA-SEL1L (labeled H+S), using 1.5 μg of these plasmids. Total cDNA was normalized to 12 μg with empty vector pcDNA. pc is α1, β2, γ2, γ2(Q390X), and pcDNA (3:3:1.5:1.5 μg). ZNS is α1, β2, γ2, γ2(Q390X), and pcDNA (3:3:1.5:1.5 μg) treated with 3 μM ZNS 24 h before harvesting. Living cells were treated with EZ-Link Sul-fo-NHS-SS-biotin to biotinylate surface proteins, which were then purified and run on polyacrylamide gels. A. Membranes were immunoblotted for γ2 (1:1000) (**A**), α1 (1:500) (**B**), or β2 (1:1000) (**C**) or ATPase (1:1000) as loading control. Protein IDVs were normalized first to the loading control and then to the WT condition (**D**–**F**). N = 5–6 separate transfections in different batches of cells. One-way ANOVA and Tukey’s test for post-hoc analysis, corrected for multiple comparisons, were used to evaluate statistical significance. * *p* < 0.05, **** *p* < 0.0001. Values are expressed as the mean ± S.E.M.

Compared with the wildtype α1β2γ2 receptors, the surface expression of the γ2 subunit was lower in the α1β2γ2/γ2(Q390X) + pcDNA (henceforth, Q390X) (0.474 ± 0.027, *p* < 0.0001). The ZNS-treated α1β2γ2/γ2(Q390X) + pcDNA condition (ZNS) showed a trend towards an increase (0.572 ± 0.017), but this was not statistically different from Q390X (*p* = 0.106) and was still lower than WT (*p* < 0.0001) (Figure 3A,D). However, for the α1 subunit, ZNS treatment rescued the reduction seen in the Q390X condition (Q390X 0.747 ± 0.057, compared to WT 1 ± 0, *p* = 0.032; compared to ZNS 0.998 ± 0.074, *p* = 0.033), such that the ZNS-treated α1β2γ2/γ2(Q390X) + pcDNA condition was indistinguishable from WT (1 ± 0 WT vs. 0.998 ± 0.074 ZNS, *p* > 0.999) (Figure 3B,E). For the β2 subunit, the Q390X condition showed lower expression than WT (0.671 ± 0.068, *p* = 0.019), and the ZNS-treated α1β2γ2/γ2(Q390X) + pcDNA condition rescued the β2 subunit expression (0.934 ± 0.117) to be not different from WT (*p* = 0.978), but this increase failed to reach statistical significance compared to the untreated Q390X condition (*p* = 0.088) (Figure 3C,F). Our data indicate that ZNS partially rescued the deleterious suppression of the γ2(Q390X) subunit on the surface trafficking of GABA_A_R subunits.

### 2.4. ZNS Selectively Increased the Wildtype γ2 Subunit but Had No Effect on Total Expression of α1 or β2 Subunits In Vitro

**Figure 4:**
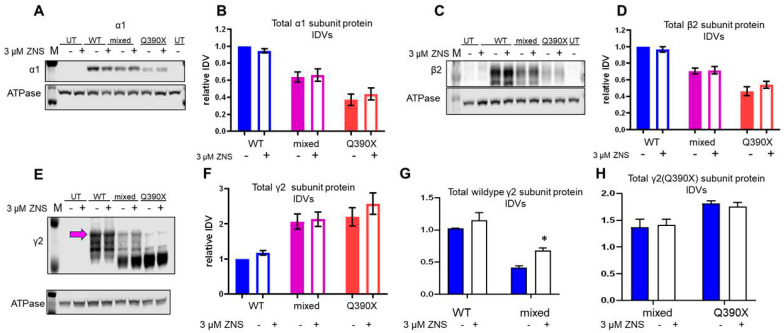
ZNS altered the total expression of the γ2 subunit, but not the α1 or β2 subunit in the mutant α1β2γ2/γ2(Q390X) receptors. (**A**–**H**). HEK293T cells were transfected with α1, β2, and γ2 (1 μg each per 60 mm dish) (WT); α1, β2, and mixed γ2 and γ2(Q390X) (1:1:0.5:0.5); or α1, β2, and γ2(Q390X) (1:1:1) (mutant). Untransfected cells were used as controls (con). Here, 24 h before harvesting, 3 μM ZNS or vehicle was applied. IDVs were normalized to ATPase (1:1000) and then to vehicle-treated WT. The membranes were immunoblotted for α1 (1:500) (**A**) or β2 (1:1000) (**C**) or γ2 (1:1000) subunit (**E**) antibodies. In (**B**,**D**,**F**,**G**,**H**), protein IDVs were normalized first to the loading control and then to the WT condition. (**A**) Immunoblot for α1 (1:500) and graph of normalized IDVs. (**B**) Immunoblot for β2 (1:1000) and graph of normalized IDVs. (**C**) Immunoblot for γ2 (1:1000) and graph of normalized IDVs. Quantification is for all γ2 signals: wildtype γ2 at 45 kDa, mutant γ2 at 39 kDa, and dimers between 80 and 100 kDa. (**D**) Graph of normalized IDVs of only monomeric wildtype γ2 at 45 kDa. (**B**,**D**,**F**,**G**,**H**) Normalized α1 (**B**), β2 (**D**), the total γ2 (**F**), the total wildtype γ2 (**G**), or the total γ2(Q390X) subunit protein (**H**) IDVs were plotted. In (**F**,**G**,**H**), the purple arrow-pointed band was not included as it is likely nonspecific. For (**H**), N = 7–8 separate transfections. Two-way ANOVA and Šídák’s multiple comparisons, examining simple effects within drug treatments, were used to evaluate statistical significance. * *p* < 0.05. Values are expressed as the mean ± S.E.M.

The increased surface expression of the α1 and γ2 subunits could be due to the increased total subunit protein expression. We then transfected HEK293T cells with the recombinant GABA_A_ receptor α1, β2, and γ2 cDNA to form the wildtype (α1β2γ2), mixed (α1β2γ2/γ2(Q390X), or mutant (α1β2γ2(Q390X)) receptors at a cDNA ratio of 1:1:1 for the wildtype; 1:1:0.5:0.5 for the mixed, and 1:1:1 for the Q390X mutant condition. The cells were treated with ZNS (3 μM) for 24 h as this concentration is identified as the most optimal one, while some cell loss was observed at higher concentrations. It is interesting that total α1 subunit was unchanged in all conditions (WT: 1 ± 0 vehicle vs. 0.946 ± 0.023 ZNS, *p* = 0.875; mixed: 0.639 ± 0.060 vehicle vs. 0.661 ± 0.073 ZNS, *p* = 0.990; mutant: 0.371 ± 0.066 vehicle vs. 0.437 ± 0.072 ZNS, *p* = 0.793) (Figure 4A,B). The β2 subunit also had no differences (WT: 1 0 vehicle vs. 0.966 ± 0.034 ZNS, *p* = 0.907; mixed: 0.705 ± 0.036 vehicle vs. 0.715 ± 0.046 ZNS, *p* = 0.997; mutant: 0.461 ± 0.054 vehicle vs. 0.539 ± 0.044 ZNS, *p* = 0.433) (Figure 4B,D).

Next, expression of the γ2 subunit after ZNS (3 μM, 24 h) treatment was evaluated. The data were analyzed for wildtype γ2 monomers alone, γ2(Q390X) monomers alone, and for total γ2 subunits, which refers to the sum of wildtype and mutant monomers and γ2 dimers. For total γ2 subunits, no differences were seen after ZNS treatment (WT: 1 ± 0 vehicle vs. 1.177 ± 0.067 ZNS, *p* = 0.907; mixed: 2.064 ± 0.216 vehicle vs. 2.136 ± 0.206 ZNS, *p* = 0.993; mutant: 2.203 ± 0.258 vehicle vs. 2.575 ± 0.308 ZNS, *p* = 0.505) (Figure 4E,F). Interestingly, in the HEK293T cells transfected with the wildtype GABA_A_R condition, a slight increase in the wildtype γ2 subunit was observed with ZNS treatment, in contrast to the effects of HRD1 overexpression (WT: 1 ± 0 vehicle vs. 1.150 ± 0.12 ZNS, *p* = 0.514; mixed: 0.41 ± 0.03 vehicle vs. 0.681 ± 0.04 ZNS, *p* = 0.0439) (Figure 4D). ZNS treatment did not change γ2(Q390X) subunit expression in the mutant conditions (mixed: 1.37 ± 0.15 vehicle vs. 1.41 ± 0.11 ZNS, *p* = 0.993; mutant: 1.81 ± 0.05 vehicle vs. 1.75 ± 0.08 ZNS, *p* = 0.976) (Figure 4E).

### 2.5. ZNS Upregulated the Expression of HRD1 Expression

**Figure 5:**
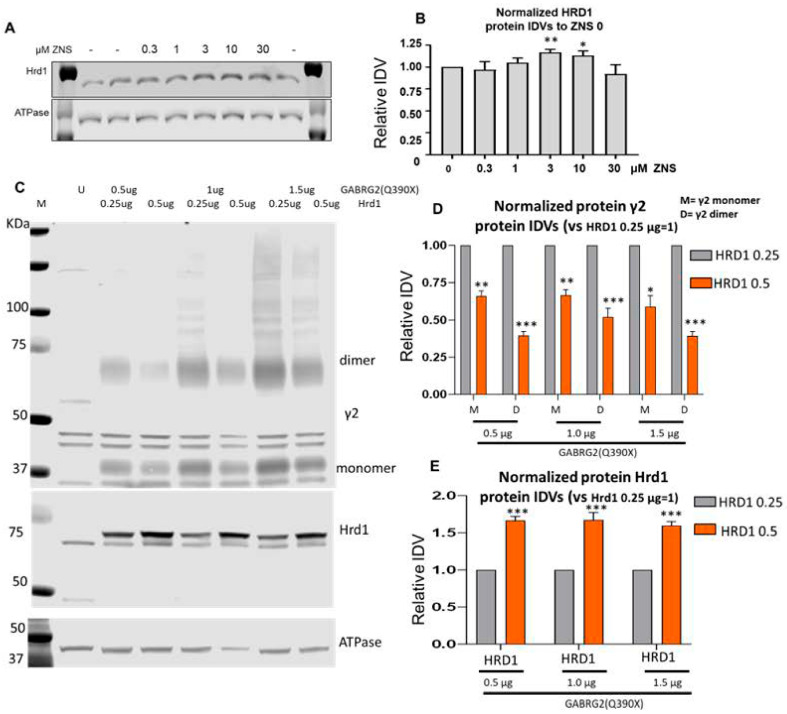
HRD1 dose-dependently enhanced degradation of the mutant γ2(Q390X) subunits. (**A**,**B**) Untransfected HEK293T cells were treated with 0.3, 1, 3, 10, or 30 μM ZNS or vehicle 48 h after passaging and 24 h before harvesting. (**A**) The SDS-PAGE membrane was immunoblotted for HRD1 (1:1000). (**B**) HRD1 IDVs were normalized to the average of all vehicle-treated dishes (ZNS 0 µM). This average was taken as 1. (**C**–**E**) HEK293T cells were transfected with γ2(Q390X) (0.5, 1, and 1.5 µg) with different HRD1 cDNA amounts. The total amount of cDNA in each condition was normalized with the vector pcDNA. The membranes were immunoblotted with an anti-γ2 subunit or HRD1 antibody. ATPase was used as a loading control. (**D**) The γ2(Q390X) subunit protein (**D**) or HRD1 (**E**) in cells cotransfecting HRD1 0.5 µg was normalized to that of HRD1 0.25 µg. For (**A**,**B**), N = 5. For (**C**–**E**), N = 6 separate transfections. Two-way ANOVA and Šídák’s multiple comparisons were used to evaluate statistical significance. In (**B**), * *p* < 0.05; ** *p* < 0.01 vs. ZNS 0 (untreated). In (**D**,**E**), * *p* < 0.05; ** *p* < 0.01; ***p < 0.001 vs. HRD1 0.25 µg. Values are expressed as the mean ± S.E.M.

Previous studies suggest that ZNS can increase HRD1 expression [51,53]. Because overexpressing the GABA_A_ receptors in the transfected cells could overwhelm the protein quality control, we thus first treated the HEK293T cells without overexpressing the GABA_A_R with varying doses of ZNS (0.3–30 μM) for 24 h. We observed a significant cell loss in the cells treated with 30 μM. Compared with vehicle-treated cells, concentrations of 3 μM and 10 μM had a small but significant increase in HRD1 expression. (Figure 5A,B). However, we failed to observe the upregulation of HRD1 in the cells expressing the wildtype α1β2γ2 or the mutant α1β2γ2(Q390X) receptors. This suggests that overexpressing the recombinant GABA_A_ receptors may dampen HRD1 induction by ZNS.

#### 2.5.1. HRD1 Dose-Dependently Degraded the Mutant γ2(Q390X) Subunit Protein in Both Monomers and Dimers

We next coexpressed HRD1 and the mutant γ2(Q390X) subunit with different cDNA amounts ranging from 0.5 µg to 1.5 µg (Figure 5C–E). We compared the effect of HRD1 by transfecting 0.25 µg or 0.5 µg HRD1 cDNA with three different doses of γ2(Q390X) cDNAs (0.5 µg, 1 µg, and 1.5 µg). The γ2(Q390X) subunit protein was reduced to a greater degree with a higher amount of HRD1 cDNA at all three γ2(Q390X) concentrations, for both monomers and dimers (for monomer: 0.66 for 0.5 µg of γ2 cDNAs; 0.67 for 1.0 µg of γ2 cDNAs, and 0.59 for 1.5 µg of γ2 cDNAs; for dimer: 0.39 for 0.5 µg of γ2 cDNAs; 0.52 for 1.0 µg of γ2 cDNAs, and 0.39 µg for 1.5 µg when compared with HRD1 0.25 µg, which is arbitrarily taken as 1) (Figure 5D). Interestingly, the magnitude of reduction of γ2 subunit protein is larger in dimer compared with that in monomer in all three concentrations. Consistently, knockdown of HRD1 increased the mutant γ2(Q390X) subunit expression (Supplementary Figure S2), suggesting the essential role of HRD1 in degrading the γ2 subunit in the ER.

#### 2.5.2. ZNS Reduced Seizures in the *Gabrg2^+/Q390X^* Mice

**Figure 6:**
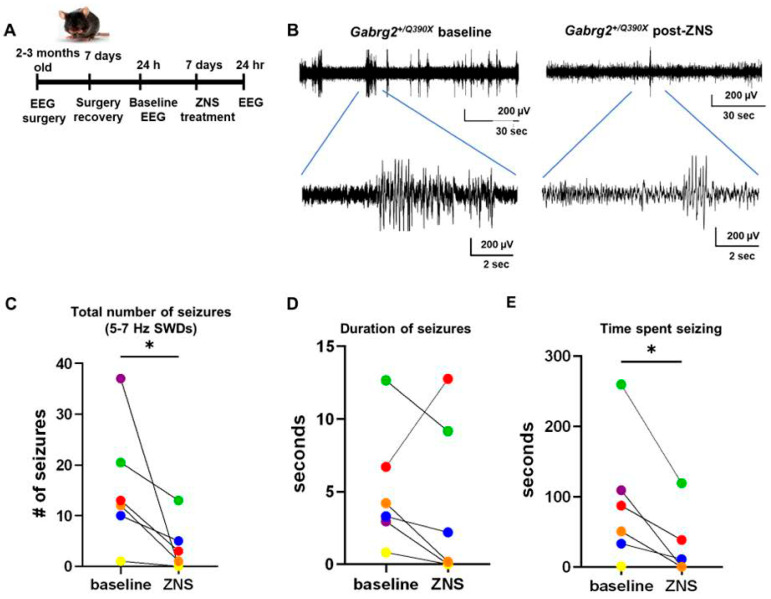
ZNS reduced seizures in *Gabrg2^+/Q390X^* mice. (**A**) Schematic showing EEG headmount affixation and recordings. (**B**) Representative traces from a *Gabrg2^+/Q390X^* mouse experiencing a 5–7 Hz spike-and-wave discharge (SWD) at baseline and after 7 days of ZNS treatment (20 mg/kg/day, administered intraperitoneally). A 60 s trace is zoomed in on a 10 s window. (**C**) Total number of 5–7 Hz SWDs in 24 h, during baseline and after ZNS treatment. (**D**) Average duration of 5–7 SWD events, during baseline and after ZNS treatment. (**E**) Total time spent seizing in 5–7 Hz SWDs in 24 h, during baseline and after ZNS treatment. N = 3 female heterozygous *Gabrg2^+/Q390X^* mice. One-tailed paired *t*-tests were used to determine significance. * *p* < 0.05 vs. baseline.

We have extensively characterized the *Gabrg2^+/Q390X^* mouse model [21]. The mouse recapitulates the major phenotype of human patients and exhibits generalized tonic–clonic seizures, increased mortality, and impaired cognition [21]. *Gabrg2^+/Q390X^* mice have spontaneous seizures beginning around P19, including absence seizures and generalized tonic–clonic seizures [21]. Thus, based on our promising in vitro results showing that ZNS normalizes GABA_A_R expression, we speculated that ZNS would help reduce seizures in Dravet syndrome caused by the *GABRG2(Q390X)* mutation. The *Gabrg2^+/Q390X^* mice were subjected to headmount affixation followed by 7 days recovery before EEG recording for baseline EEG evaluation. The mice were then administered ZNS (20 mg/kg/day for 7 days), followed by EEG recording again for drug efficacy (Figure 6A). Compared to baseline recordings, mice 3 months of age that were treated with ZNS had fewer 5–7 Hz spike-and-wave discharges (SWDs) during a 24 h period (15.58 ± 4.987 baseline vs. 3.667 ± 2.028 ZNS, *p* = 0.0358), and the total time spent seizing was reduced (90.04 ± 37.34 s baseline vs. 28.10 ± 19.19 ZNS, *p* = 0.0176) (Figure 6C–E). At this dose of ZNS, the duration of SWD events that did occur was not altered (5.106 ± 1.700 s baseline vs. 4.050 ± 2.260 ZNS, *p* = 0.2584) (Figure 6D). Our findings suggest that ZNS alone can reduce seizures in *Gabrg2^+/Q390X^* mice. Future studies with a parallel comparison between ZNS and standard anti-epileptic treatments for Dravet syndrome, such as clobazam, will provide more insights into the efficacy of ZNS in Dravet syndrome and epilepsy in general.

### 2.6. ZNS Increased the γ2 Subunit of the Wildtype Allele in the Hippocampus of the Mutant Gabrg2^+/Q390X^ Mice

**Figure 7:**
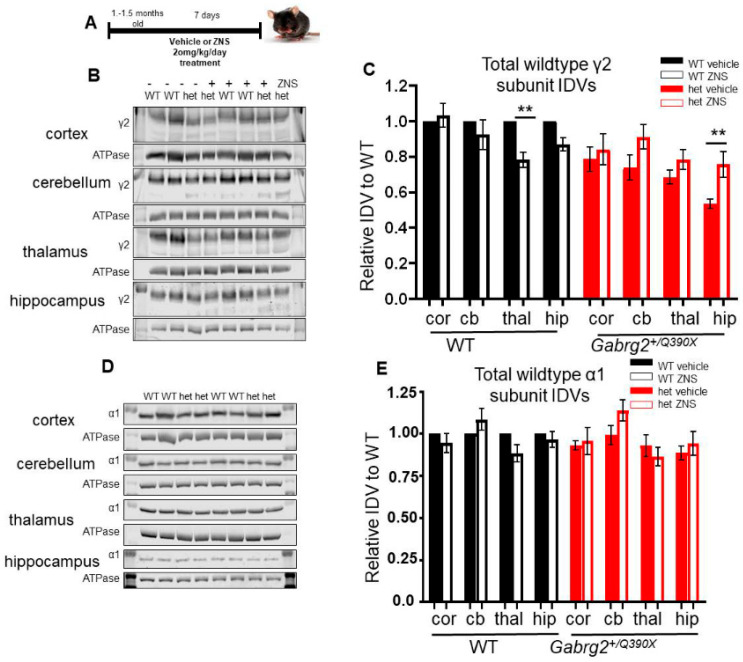
ZNS selectively altered γ2 subunit expression in *Gabrg2^+/Q390X^* mice. (**A**) Schematic depicting the experimental protocol for ZNS administration. (**B**–**E**) *Gabrg2^+/Q390X^* mice and wildtype littermates of 1–1.5 months old were treated with 20 mg/kg ZNS or an equal volume of DMSO/saline vehicle, with daily intraperitoneal injections for 7 days. Brains were dissected, and lysates of the somatosensory cortex (cor), cerebellum (cb), thalamus (thal), and hippocampus (hip) were used for SDS-PAGE. The membranes after SDS-PAGE were immunoblotted for γ2 (1:1000) (**B**) or α1 (1:500) (**D**) subunit antibodies. Only the band of the wildtype γ2 subunit was quantified, as the γ2(Q390X) subunit is not always visible. In (**C**, **E**), specific protein IDVs were normalized to the loading control, ATPase (1:1000), and then to a paired vehicle-treated wildtype animal. N = 6–8 animals. Two-way ANOVA and Šídák’s multiple comparisons, examining simple effects within drug treatments, were used to evaluate statistical significance. ** *p* < 0.01. Values are expressed as the mean ± S.E.M.

We next investigated if the reduction of seizures in *Gabrg2^+/Q390X^* mice by ZNS treatment was due to the rescue of the expression of GABA_A_R subunits. Shortly after seizure onset (1–1.5 months of age), *Gabrg2^+/Q390X^* mice and wildtype littermates were treated with ZNS (20 mg/kg/day for 7 days) (Figure 7A). Following treatment, brain lysates were analyzed for total expression of α1, β2, and γ2 subunits. Multiple brain regions were examined: the somatosensory cortex and thalamus were chosen for their role in seizures via the thalamocortical circuit, and the cerebellum and hippocampus were chosen to enable comparisons to our prior study [21,54].

Interestingly, the ZNS treatment differentially affected γ2 subunit expression in both wildtype and heterozygous animals. In wildtype animals, γ2 subunit expression decreased by 10% in the thalamus but was unchanged in other regions (cortex: 1 ± 0 vehicle vs. 1.033 ± 0.076 ZNS, *p* = 0.934; cerebellum: 1 ± 0 vehicle vs. 0.924 ± 0.084 ZNS, *p* = 0.698; thalamus: 1 ± 0 vehicle vs. 0.7844 ± 0.042 ZNS, *p* = 0.0044; hippocampus: 1 ± 0 vehicle vs. 0.871 ± 0.037 ZNS, *p* = 0.146) (Figure 7B,C). But in heterozygous mice, the wildtype form of the γ2 subunit exhibited 41% increased expression in the hippocampus compared to vehicle-treated mice, while other regions were unaffected (cortex: 0.786 ± 0.068 vehicle vs. 0.841 ± 0.086 ZNS, *p* = 0.831; cerebellum: 0.739 ± 0.070 vehicle vs. 0.912 ± 0.069 ZNS, *p* = 0.176; thalamus: 0.685 ± 0.039 vehicle vs. 0.784 ± 0.057 ZNS, *p* = 0.244; hippocampus: 0.536 ± 0.026 vehicle vs. 0.757 ± 0.073 ZNS, *p* = 0.008).

In ZNS-treated *Gabrg2^+/Q390X^* mice, the mutant γ2(Q390X) subunit demonstrated an increase or a trend of increase in the four brain regions: 31% in the cerebellum (cortex: 1 ± 0 vehicle vs. 1.452 ± 0.302 ZNS, *p* = 0.315; cerebellum: 1 ± 0 vehicle vs. 1.314 ± 0.072 ZNS, *p* = 0.0003; thalamus: 1 ± 0 vehicle vs. 1.262 ± 0.078 ZNS, *p* = 0.097; hippocampus: 1 ± 0 vehicle vs. 1.285 ± 0.160 ZNS, *p* = 0.301)

### 2.7. ZNS Did Not Change Total Expression of α1 or β2 Subunits in Mice

In contrast to the γ2 subunits, the partnering subunits were unchanged by ZNS treatment for either genotype (*Gabrg2^+/Q390X^* or wildtype littermates). For the α1 subunit, ZNS treatment resulted in no change in any brain region (cortex: WT: 1 ± 0 vehicle vs. 0.944 ± 0.057 ZNS, *p* = 0.761; het: 0.931 ± 0.029 vehicle vs. 0.956 ± 0.081 ZNS, *p* = 0.944; cerebellum: WT: 1 ± 0 vehicle vs. 1.085 ± 0.085 ZNS, *p* = 0.526; het: 0.992 ± 0.056 vehicle vs. 1.138 ± 0.063 ZNS, *p* = 0.168; thalamus: WT: 1 ± 0 vehicle vs. 0.883 ± 0.051 ZNS, *p* = 0.227; het: 0.930 ± 0.065 vehicle vs. 0.863 ± 0.055 ZNS, *p* = 0.603; hippocampus: WT: 1 ± 0 vehicle vs. 0.966 ± 0.047 ZNS, *p* = 0.876; het: 0.866 ± 0.041 vehicle vs. 0.942 ± 0.070 ZNS, *p* = 0.702) (Figure 7D,E). Similarly, the β2 subunit was universally unchanged (cortex: WT: 1 ± 0 vehicle vs. 1.102 ± 0.108 ZNS, *p* = 0.621; het: 0.983 ± 0.082 vehicle vs. 1.015 ± 0.065 ZNS, *p* = 0.954; cerebellum: WT: 1 ± 0 vehicle vs. 1.173 ± 0.111 ZNS, *p* = 0.553; het: 1.072 ± 0.109 vehicle vs. 1.383 ± 0.160 ZNS, *p* = 0.164; thalamus: WT: 1 ± 0 vehicle vs. 0.988 ± 0.069 ZNS, *p* = 0.986; het: 0.960 ± 0.069 vehicle vs. 0.937 ± 0.047 ZNS, *p* = 0.951; hippocampus: WT: 1 ± 0 vehicle vs. 1.211 ± 0.186 ZNS, *p* = 0.404; het: 0.944 ± 0.111 vehicle vs. 1.086 ± 0.037 ZNS, *p* = 0.835) (Figure 7D,E and Supplementary Figure S3A,B).

### 2.8. The Gabrg2^+/Q390X^ Mice Had Increased ER Chaperones Like BiP, which Had Differential Response to ZNS Compared to the Wildtype Mice

**Figure 8:**
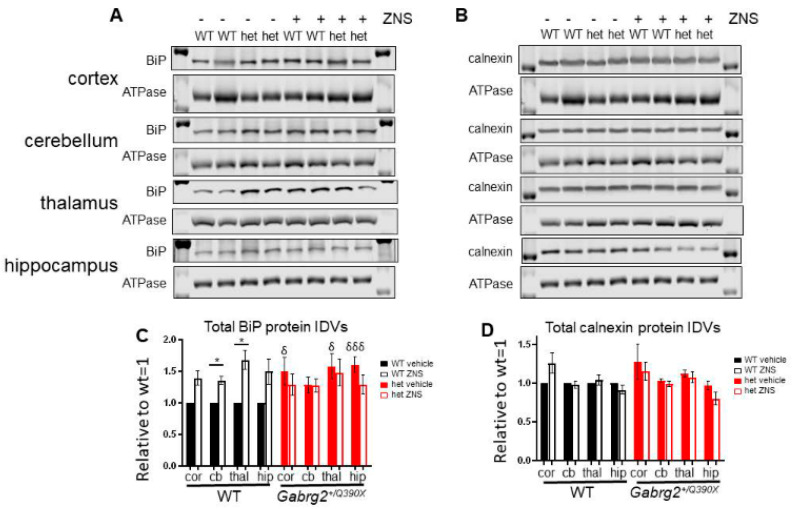
BiP was upregulated in the mutant *Gabrg2^+/Q390X^* mice and had a differential response to ZNS compared to the wildtype mice. (**A**–**D**) The wildtype and *Gabrg2^+/Q390X^* mouse littermates at post-natal day 30–45 were treated with 20 mg/kg ZNS or an equal volume of DMSO/saline vehicle, with daily intraperitoneal injections for 7 days. Brains were dissected, and lysates of the somatosensory cortex (cor), cerebellum (cb), thalamus (thal), and hippocampus (hip) were used for SDS-PAGE. The membranes after SDS_PAGE were immunoblotted for BiP (1:500) (**A**,**C**) or Calnexin (1:500) (**B**,**D**). In (**C**,**D**), specific protein IDVs were normalized to the loading control, ATPase (1:1000), and then to a paired vehicle-treated wildtype animal. (**C**,**D**) N = 6–8 animals. Two-way ANOVA and Šídák’s multiple comparisons. * *p* < 0.05 vs. wt ZNS of the same brain region; δ *p* <0.05; δδδ *p* < 0.001 vs. wt vehicle of the same brain region. Values are expressed as the mean ± S.E.M.

We have previously reported that the γ2(Q390X) subunit causes ER stress [18,21], which could be relieved by ZNS as it has been reported to prevent ER stress [49,50,51,52]. Therefore, we investigated the ER-localized molecular chaperones BiP/GRP78 and calnexin, which have been shown to interact with GABA_A_R subunits [26,37]. Compared with the wildtype, BiP is upregulated in *Gabrg2^+/Q390X^* mice, but the extent of the increase varied between different brain regions (cortex: WT 1 ± 0 vs. het 1.496 ± 0.219, *p* = 0.0470; cerebellum: WT 1 ± 0 vs. het 1.282 ± 0.128, *p* = 0.0522; thalamus: WT 1 ± 0 vs. het 1.575 ± 0.202, *p* = 0.0172; hippocampus: WT 1 ± 0 vs. het 1.605 ± 0.123, *p* = 0.0006) (Figure 8A,C). Interestingly, ZNS treatment only increased BiP expression in the wildtype but not in heterozygous littermates (Figure 8A,C) (WT: 1 ± 0 vehicle vs. 1.352 ± 0.059 ZNS, *p* = 0.023; het: 1.282 ± 0.128 vehicle vs. 1.272 ± 0.111 ZNS, *p* = 0.997) and thalamus (WT: 1 ± 0 vehicle vs. 1.680 ± 0.155 ZNS, *p* = 0.023; het: 1.575 ± 0.202 vehicle vs. 1.480 ± 0.211 ZNS, *p* = 0.914). Hippocampus showed a strong trend that failed to reach statistical significance (WT: 1 ± 0 vehicle vs. 1.495 ± 0.200 ZNS, *p* = 0.064; het: 1.605 ± 0.123 vehicle vs. 1.291 ± 0.148 ZNS, *p* = 0.300). Only in the cortex, there was no difference for either genotype (WT: 1 ± 0 vehicle vs. 1.389 ± 0.114 ZNS, *p* = 0.149; het: 1.496 ± 0.219 vehicle vs. 1.285 ± 0.163 ZNS, *p* = 0.546).

We also determined the expression of another ER chaperone, calnexin. However, calnexin expression was not altered in any brain region examined for either genotype (cortex: WT: 1 ± 0 vehicle vs. 1.258 ± 0.131 ZNS, *p* = 0.376; het: 1.279 ± 0.299 vehicle vs. 1.158 ± 0.117 ZNS, *p* = 0.799; cerebellum: WT: 1 ± 0 vehicle vs. 0.983 ± 0.048 ZNS, *p* = 0.937; het: 1.037 ± 0.026 vehicle vs. 0.991 ± 0.038 ZNS, *p* = 0.630; thalamus: WT: 1 ± 0 vehicle vs. 1.040 ± 0.065 ZNS, *p* = 0.866; het: 1.123 ± 0.046 vehicle vs. 1.078 ± 0.070 ZNS, *p* = 0.838; hippocampus: WT: 1 ± 0 vehicle vs. 0.917 ± 0.055 ZNS, *p* = 0.569; het: 0.971 ± 0.051 vehicle vs. 0.805 ± 0.080 ZNS, *p* = 0.122) (Figure 8B,D). Interestingly, HRD1 and Sel1L expression were unchanged (Supplementary Figure S4).

## 3. Discussion

### 3.1. The γ2(Q390X) Subunit Impairs the Wildtype GABA_A_R Expression and Disturb ER Protein Homeostasis

We have previously characterized the γ2(Q390X) mutant protein. This mutant protein has a loss of function and reduces the total expression of the partnering GABA_A_R subunits α1 and β2. This reduction is accompanied by a decrease in GABA_A_R surface expression, which results in diminished amplitude of miniature inhibitory post-synaptic currents (mIPSCs) and of GABA-evoked currents [16,18,21]. In addition to impairing the function of the GABA_A_ receptor channels, the mutant protein disturbs ER chaperone proteins and causes ER stress [22].

### 3.2. The E3 Ligase HRD1 Facilitates Degradation of the Mutant γ2(Q390X) Subunit

Our previous studies have demonstrated that epilepsy-associated mutant GABA_A_R subunits are degraded in part through ERAD [55,56]. The ERAD component HRD1, an E3 ubiquitin ligase, has been shown to ubiquitinate the α1 subunit, thereby marking the α1 subunit for degradation via the ubiquitin-proteasome system [26,37]. As the α1 and γ2 subunits share 44% sequence identity, it was probable that HRD1 may also be capable of targeting the γ2 subunit. Indeed, pharmacological inhibition of HRD1 not only slowed the degradation of an α1 mutant subunit that is normally rapidly degraded, it also elevated the expression of two epilepsy-associated missense γ2 subunit mutations [37]. We have compared the effect of HRD1 with other E3 ligases, including NEDD4L, UBE3A, RNF34, and SEL1L, and found that HRD1 most efficiently degraded the ER-bound wildtype and the mutant γ2(Q390X) subunit.

### 3.3. Enhancing Degradation of the Mutant γ2(Q390X) Subunit Could Relieve the Dominant Negative Suppression on the Wildtype γ2 Subunits

The *GABRG2(Q390X)* mutation dominant-negatively suppresses the wildtype subunits and exacerbates disease phenotype based on comparison of *Gabrg2^+/Q390X^* and *Gabrg2^+/−^* mice [21]. Our findings indicate that decreasing the γ2(Q390X) subunit protein via HRD1-related mechanisms may reduce ER retention of wildtype γ2 subunit, resulting in more efficient trafficking of GABA_A_Rs to the cell surface and thus increasing GABA_A_R channel function. Although increased HRD1 activity may reduce the total amount of wildtype γ2 further, the increase in surface trafficking of functional receptors by removing γ2(Q390X) may offset the decrease in the total γ2 subunit. *Gabrg2^+/−^* mice have a much milder phenotype than *Gabrg2^+/Q390X^* mice, suggesting less efficient trafficking of the wildtype subunits in the presence of the γ2(Q390X) subunit [21,57].

### 3.4. ZNS Partially Restored Surface Trafficking of the Wildtype GABA_A_R Subunits and Reduced Seizures in the Gabrg2^+/Q390X^ Mice

ZNS is an approved antiseizure drug, and its antiseizure mechanism is complex [58,59]. In addition to its direct effect on ion channels via altering the fast inactivation threshold of voltage-dependent sodium channels and reducing sustained high-frequency repetitive firing of action potentials [60], it has been reported that ZNS can reduce Cx43 expression and cell–cell coupling in the astrocyte-microglia co-culture, suggesting additional anti-seizure effects of ZNS on modifying the disruption of glial gap-junctional communication under inflammatory conditions [61]. This is not surprising, as gap junctions between astrocytes play an important role in the development of epilepsy and extracellular epileptic electrical activity in vitro [62]. Our findings suggest that ZNS can increase the surface expression of GABA_A_Rs in the cells expressing the mutant α1β2γ2/γ2(Q390X) receptors. Both α1 and γ2 subunits were increased, suggesting the functional rescue of the receptor. At the total level, we only observed an increase in the wildtype γ2 subunit in the α1β2γ2/γ2(Q390X) receptors, suggesting the increased biogenesis of the wildtype γ2 subunit. The increased α1 subunit at the cell surface suggests more efficient trafficking with ZNS treatment. This is at least partially due to the fact that ZNS can reduce ER stress and moderately enhance HRD1.

The wildtype γ2 subunit was selectively upregulated in the *Gabrg2^+/Q390X^* mouse. Compared with other brain regions, the upregulation of the wildtype γ2 subunit in the hippocampus was most prominent. This suggests the upregulation of the γ2 subunit in the *Gabrg2^+/Q390X^* mouse is brain region-dependent. This is likely due to the abundance of the specific subunit expression. Interestingly, ZNS downregulated the γ2 subunit in the wildtype mice, suggesting that the response of GABA_A_R expression to ZNS is genotype-dependent.

Our data indicate that ZNS, at the dose of 20 mg/kg/day, was effective in reducing the number of spontaneous 5–7 Hz SWD seizures in 3-month-old *Gabrg2^+/Q390X^* mice. Furthermore, the total time spent seizing was 69% lower compared with untreated ones. Thus, ZNS may be useful for *GABRG2(Q390X)*-associated Dravet syndrome. In addition to seizures, it will be interesting to evaluate other Dravet-associated phenotypes in ZNS-treated *Gabrg2^+/Q390X^* mice, such as anxiety, social abnormalities, and spatial memory [21].

### 3.5. ZNS Treatment Had Differential Effects on the Upregulation of the ER Chaperone BiP

Our data indicate that ZNS differentially modulates the expression of chaperone protein in the endoplasmic reticulum between the wildtype and the *Gabrg2^+/Q390X^* mice. Since ZNS only moderately increased HRD1 in the cell model but not in the mouse model, and it upregulated BiP in the wildtype mice, it is possible that ZNS increased GABA_A_R trafficking via a wide network of ER proteins. Previous studies have suggested a protective role for ZNS against ER stress [49,50,51,52]. Removal of ER-retained mutant proteins could relieve ER stress and facilitate protein forward trafficking. We identified that BiP was increased in *Gabrg2^+/Q390X^* mice. There are many proteins involved in the ER stress and unfolded protein response pathways, such as ATF6, PERK, IRE1α, and CHOP, so these additional factors may also be involved and should be investigated to obtain a more comprehensive understanding of the interaction between ZNS, GABA_A_R subunits, and protein trafficking.

Our findings provide critical insights into how the ER chaperones in the *Gabrg2^+/Q390X^* mouse have changed compared to the wildtype ones. Consequently, the response to ZNS is different in wildtype and mutant mice. These changes may not be easily identified in mice. In HEK293T cells, the protein quality control could be overwhelmed due to the protein overexpression. BiP was strongly upregulated by ZNS, but, interestingly, only in wildtype animals. This disparity suggests that the ER stress pathway is altered in *Gabrg2^+/Q390X^* mice, as they did not respond in this manner to ZNS. Indeed, BiP expression is approximately 50% higher in *Gabrg2^+/Q390X^* mice compared to littermates in all four brain regions examined. This is in line with our prior in vitro findings that the γ2(Q390X) protein is associated with increased expression of the ER-stress-induced pro-apoptotic factor GADD153/CHOP [18,21]. The elevated expression of BiP at baseline and lack of response to ZNS raise the possibility of a ceiling effect, wherein the molecular chaperones in the ER of neurons of *Gabrg2^+/Q390X^* mice are operating at full capacity. The γ2(Q390X) subunit may deplete the reserve capacity of protein folding pathways, such that these cells are perhaps not capable of promoting further protein quality control measures. More research is necessary to fully characterize the effects of the γ2(Q390X) subunit mutation on ER stress and proteostasis.

## 4. Materials and Methods

### 4.1. Cell Culture and Polyethyleneimine Transfection

Human embryonic kidney 293 T (HEK293T) cells were grown in Dulbecco’s Modified Eagle’s Medium (DMEM, Life Technologies Corporation, Grand Island, NY, USA) supplemented with 10% FBS and 1% penicillin/streptomycin. Then, 24 h after plating the cells, they were transfected with cDNA for wildtype rat γ2S or γ2S(Q390X) subunits (referred to hereafter simply as γ2 or γ2(Q390X) subunits, respectfully), human α1 subunit, and/or human β2 subunit. cDNA was combined with polyethyleneimine (PEI) at a ratio of 2.5 μL PEI per 1 μg cDNA. Additional cDNA included myc-tagged HRD1 and HA-tagged SEL1L, kindly shared by Dr. Nobuko Hosokawa at the Institute for Frontier Life and Medical Sciences, Kyoto University, and UBE3A-HA, kindly shared by Dr. James Sutcliffe at Vanderbilt University Medical Center. Further cDNA plasmids were purchased from Addgene (Watertown, MA, USA): HA-RNF34 (119938) and HA-NEDD4L (27000). Zonisamide (ZNS) (Tocris 2625) dissolved in dimethyl sulfoxide (DMSO) was used in varying concentrations specified in the text.

### 4.2. Immunoblot

Cells were harvested 48 h after transfection. Cells were washed with cold phosphate buffered saline (PBS) and lysed with a RIPA-PI solution containing 20 mM Tris, 20 mM EGTA, 1 mM DTT, 1 mM benzamidine, 0.01 mM PMSF, 0.005 μg/mL leupeptin, and 0.005 μg/mL pepstatin. Protein concentration was measured, and samples were subjected to standard SDS polyacrylamide gel electrophoresis (SDS-PAGE) procedures and immunoblotted. Primary antibodies used were anti-α1 1:500 (Millipore Sigma MABN489, Burlington, MA, USA), anti-β2 1:1000 (Millipore Sigma AB5561), anti-γ2 1:1000 (Synaptic Systems 224003, Goettingen Germany), anti-BiP 1:500 (BD Biosciences 610979, Franklin Lakes, NJ, USA), anti-calnexin 1:1000 (Enzo Life Sciences ADI-SPA-860-F, Farmingdale, NY, USA), anti-NEDD4L 1:1000 (Cell Signaling Technology 4013, Danvers, MA, USA), anti-UBE3A 1:1000 (Atlas Antibodies HPA039410, Bromma, Stockholms Lan, Sweden), anti-HRD1 1:1000 (Novus Biologicals NB100-2526, Centennial, CO, USA), anti-SEL1L 1:1000 (Novus Biologicals NBP2-93746), anti-HA 1:300 (Cell Signaling Technology 3724S), anti-myc (Millipore Sigma 05-724), and anti-ATPase 1:1000 (Developmental Studies Hybridoma Bank a6F). Secondary antibodies were LI-COR IRDye 680LT Goat anti-Mouse IgG Secondary Antibody (926-68020) and IRDye 800CW Goat anti-Rabbit IgG Secondary Antibody (926-32211), both 1:10,000.

Blots were imaged with an Odyssey DLx digital fluorescence scanner and LI-COR Image Studio Lite 5.2 software. Protein bands were quantified by circumscribing the band of interest and correcting for background signals. Integrated density values (IDVs) for the protein of interest were normalized to the IDV for the loading control, ATPase. The values were then normalized to loading controls and then the normalized IDV of the control lane (generally, wildtype or untreated), which was arbitrarily taken as equal to 1.

### 4.3. Biotinylation

The experiment procedure is based on our standard laboratory protocol as described [16,63]. Briefly, transfected HEK293T cells were gently washed with room temperature PBS-Ca-Mg (PBS with 0.1 mM CaCl_2_ and 1 mM MgCl_2_) and then incubated with EZ-Link Sulfo-NHS-SS-biotin (Thermo Scientific 21331, Waltham, MA, USA) in PBS-Ca-Mg. The biotinylation reaction was then quenched with 0.1 M glycine in PBS-Ca-Mg. Cells were then collected and lysed in standard RIPA-PI buffer. Biotinylated proteins were purified by incubating the cell lysates overnight with high-capacity Neutravidin agarose resin beads (Thermo Scientific 29202). After incubation, the beads were washed with RIPA-PI to remove nonbiotinylated protein, and biotinylated protein was next eluted from the beads with Laemmli sample buffer containing β-mercaptoethanol. Samples were then subjected to standard immunoblot procedures.

### 4.4. Gabrg2^+/Q390X^ Mouse Model of GEFS+ and Dravet Syndrome

The generation of the *Gabrg2^+/Q390X^* mouse was described previously [21].

### 4.5. Drug Administration and Brain Tissue Preparation in Gabrg2^+/Q390X^ Mice

*Gabrg2^+/Q390X^* mice in the C57BL/6J (Jackson Labs stock 000664, Bar Harbor, ME, USA) background were bred with wildtype C57BL/6J mice. Animals were housed in standard facilities with ad libitum food and water access. Beginning at 1–1.5 months of age, heterozygous animals and wildtype littermates, both male and female, were treated daily with vehicle or 20 mg/kg ZNS injected intraperitoneally for 7 days. ZNS (Tocris 2625, Bristol, UK) was dissolved in 10% DMSO and 90% 0.9% saline for a final concentration of 5 mg/mL. After 7 days of treatment, the mice were anesthetized with isoflurane and decapitated. The brain was removed, and the somatosensory cortex, cerebellum, thalamus, and hippocampus were dissected.

### 4.6. EEG Acquisition and Scoring

Around 2–3 months of age, male and female *Gabrg2^+/Q390X^* mice were surgically implanted with headmounts from Pinnacle Technology that have two bipolar electroencephalogram (EEG) channels and one subcutaneous nuchal electromyogram (EMG) channel (Pinnacle 8201: 2 EEG/1 EMG Mouse Headmount). After recovering from headmount surgery for 7 days, a 24 h baseline recording was acquired using EEG and EMG channels and simultaneous video. Mice were then treated daily with 20 mg/kg ZNS injected intraperitoneally for 7 days, and on the 8th day, a 24 h EEG recording was again acquired. EEGs were acquired with Sirenia Acquisition, with the sampling rate set at 400 Hz with a pre-amplifier gain of 100 Hz. EEG and EMG channels have a filter set at 25 Hz. There were two independent electrodes that were inserted into the back neck muscle to measure EMG activity, reflected by the electrical potential arising from the neuronal activation associated with muscle contraction. A high amplitude EMG reflects active movement, while a tonic EMG signal indicates quiescence or sleep.

EEGs are scored by a blinded, skilled scorer with Pinnacle 9037 Sirenia Seizure Pro software. A power analysis of the theta frequency band of 5–7 Hz was used to identify seizures, as 5–7 Hz spike-and-wave discharges (SWDs) are the mice’s correlate of human 2–4 Hz SWDs, which is representative of absence or absence-like activity in human patients. The correlation of 2–4 Hz SWDs in human patients and 5–7 Hz SWDs in mice has been reported in our previous study [64]. Suspected seizure events were manually confirmed by correlating the EEG activity and the mouse behavior from the video recording.

### 4.7. Data Analysis

The data were analyzed using GraphPad Prism 9.4 and are reported as the mean ± standard error of the mean (SEM). Overexpression of E3 ubiquitin ligases was analyzed via one-way analysis of variance (ANOVA) and post-hoc analysis using Šídák’s multiple comparisons test. For cell culture experiments overexpressing HRD1 and SEL1L, one-way ANOVA tests were performed. Each condition was compared to all other conditions using Tukey’s test for post-hoc analysis, corrected for multiple comparisons. For EEG data, paired *t*-tests were used. Simple linear regression was used to evaluate the dose–response effect of ZNS on HRD1 expression. For other cell culture and mouse experiments utilizing ZNS, two-way ANOVAs were performed, fitting a full interaction model. Post-hoc analysis was performed using Šídák’s multiple comparisons, examining simple effects within drug treatments. Statistical significance was taken as *p* < 0.05 throughout.

## 5. Conclusions

ERAD is known to be altered in some genetic diseases with epilepsy, including familial encephalopathy with neuroserpin inclusion bodies (FENIB) [65] and RNF13-associated infantile neurodegeneration [66]. Additionally, many other genetic epilepsies are associated with misfolded and/or mistrafficked proteins, such as those caused by mutations in *SLC6A1* [67,68], *STXBP1* [69,70], and *KCNQ2* [71]. Together, this points towards a possible avenue of treatment: modulation of proteostasis. Proteostasis regulators have previously been explored for other monogenetic epilepsies, such as NMDA receptor-associated epilepsies [72]. We here identified that a component of ERAD, an endogenous protein quality control pathway—specifically, the E3 ubiquitin ligase HRD1—can decrease expression of the neurotoxic γ2(Q390X) subunit. Modulation of HRD1 alone can alter GABA_A_R expression. Consistently, dinoprost (DNP) and dihydroergocristine (DHEC) inhibit HRD1, allowing mutant GABA_A_R subunits that are subject to overactive degradation to instead insert into receptors with partial functionality [37]. Our findings here support the prior study on HRD1, proteostasis regulators, and genetic epilepsies and suggest that this class of drug could be repurposed for at least a subset of epilepsies caused by mutations like *GABRG2(Q390X)*.

## Data Availability

The datasets used and/or analyzed during the current study are available from the corresponding author on reasonable request.

## Supplementary Materials

The following supporting information can be downloaded at: https://www.mdpi.com/article/10.3390/ijms25094601/s1.

## Abbreviations

DHEC	dihydroergocristine
DNP	dinoprost
EEG	electroencephalogram
EMG	electromyogram
ER	endoplasmic reticulum
ERAD	ER-associated degradation
FENIB	familial encephalopathy with neuroserpin inclusion bodies
GABA	γ-aminobutyric acid
GABAAR	GABA receptor type A
SEM	standard error of the mean
SWD	spike-and-wave discharge
ZNS	zonisamide
