# Supplementary material for: Modulating Endoplasmic Reticulum Chaperones and Mutant Protein Degradation in GABRG2(Q390X) Associated with Genetic Epilepsy with Febrile Seizures Plus and Dravet Syndrome
Source: Int J Mol Sci. 2024 Apr 23;25(9):4601. doi: 10.3390/ijms25094601 (PMC11083348; doi:10.3390/ijms25094601)

Supplementary Figure 1: Differential effect of E3 ubiquitin ligases on expression of the γ2 or γ2(Q390X) subunits

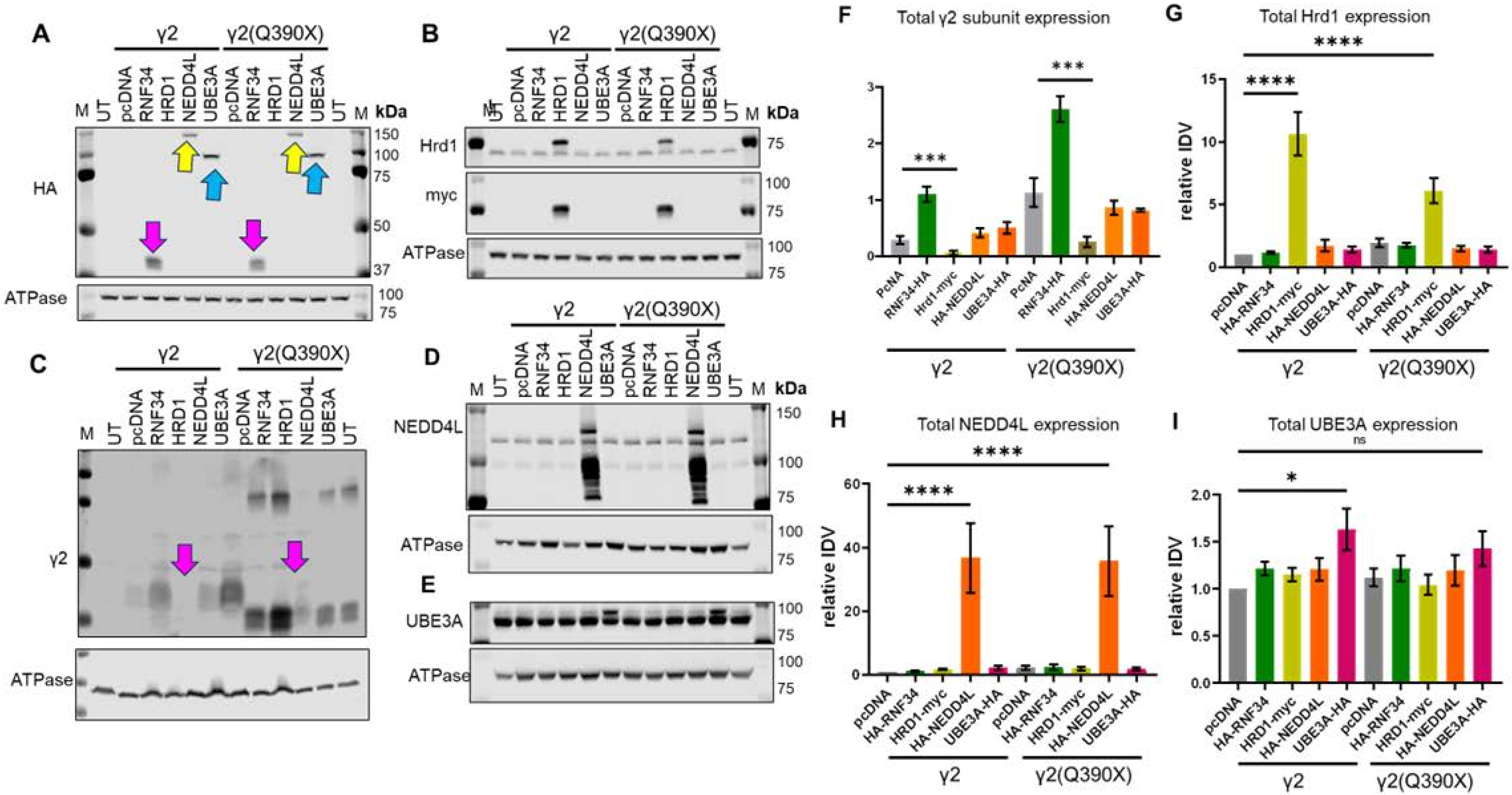

Supplementary Figure 2: ERAD component HRD1 modulate the degradation of the mutant γ2(Q390X) subunit protein

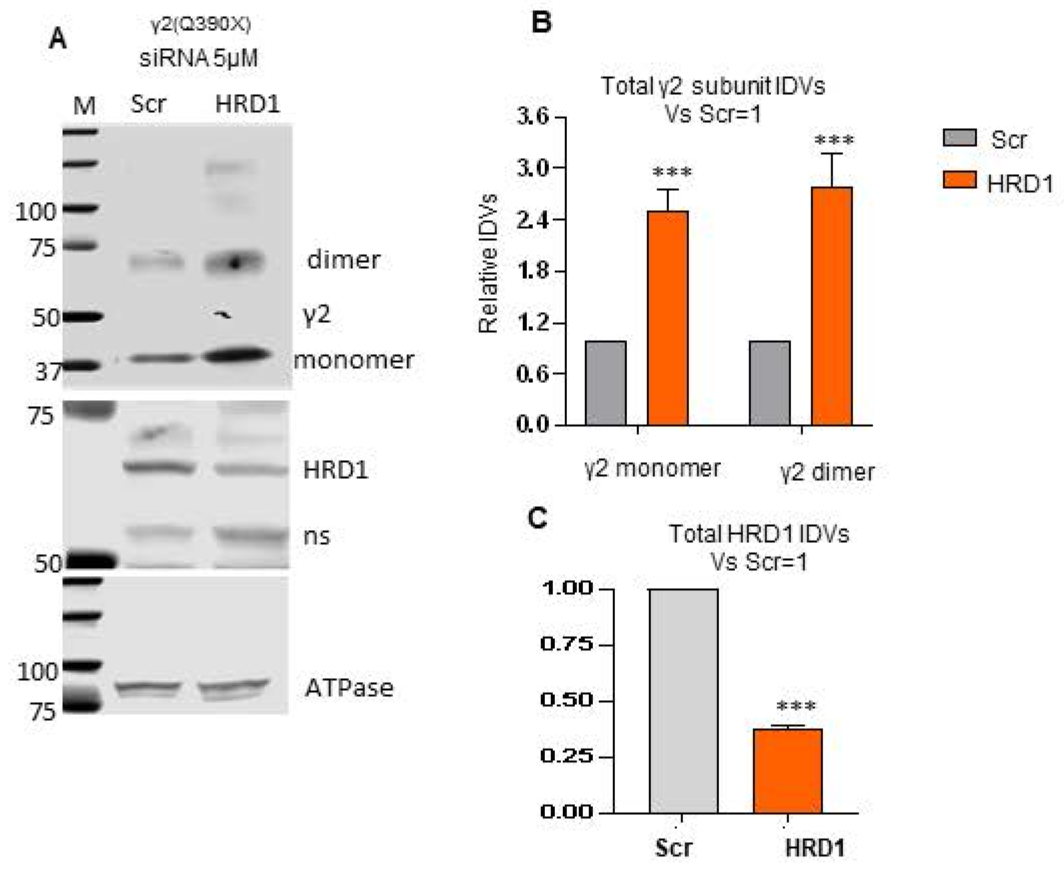

Supplementary Figure 3: ZNS did not altered β2 subunit expression in *Gabrg2*$^{+/Q390X}$ mice

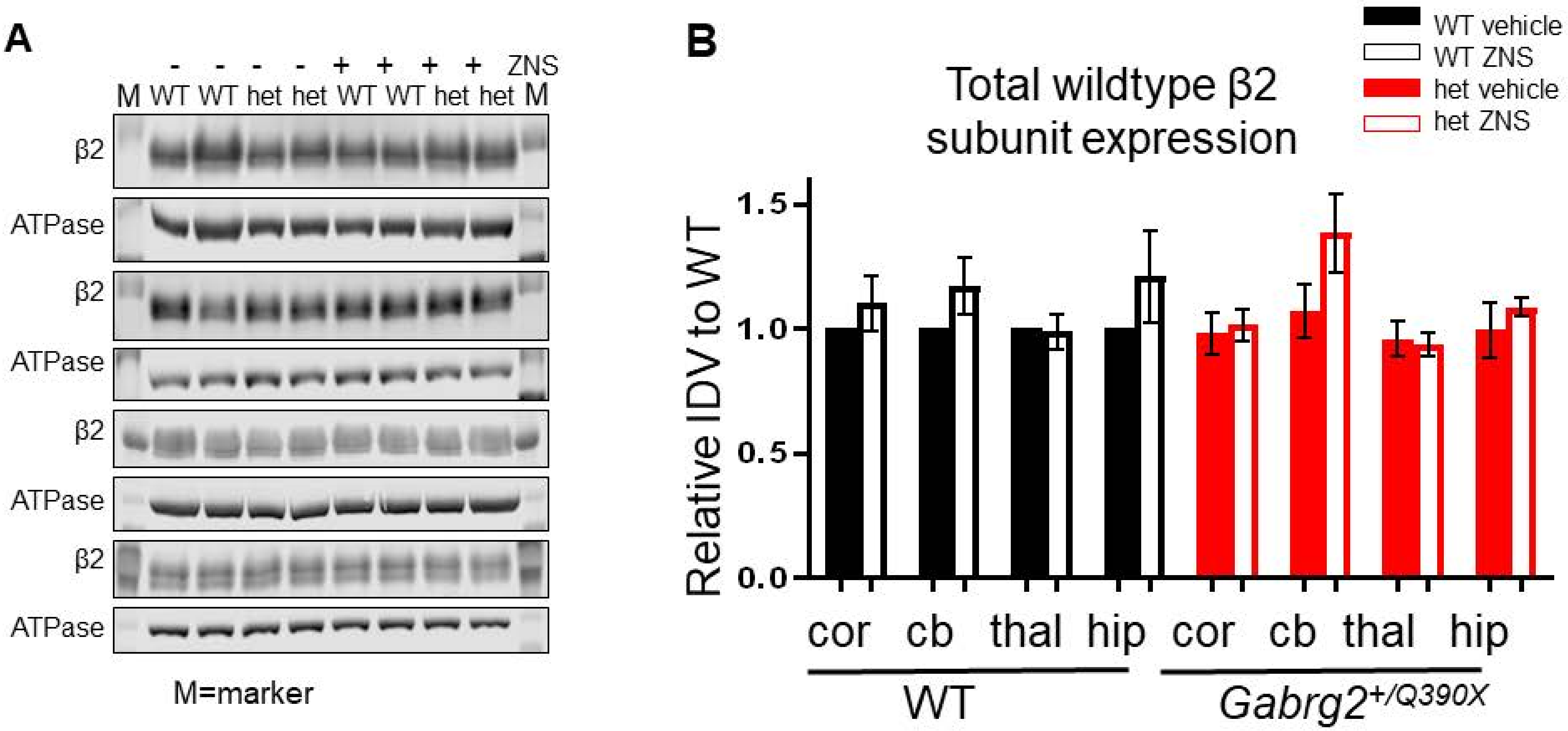

Supplementary Figure 4: ZNS did not alter Hrd1 or Sel1L expression in *Gabrg2*$^{+/Q390X}$

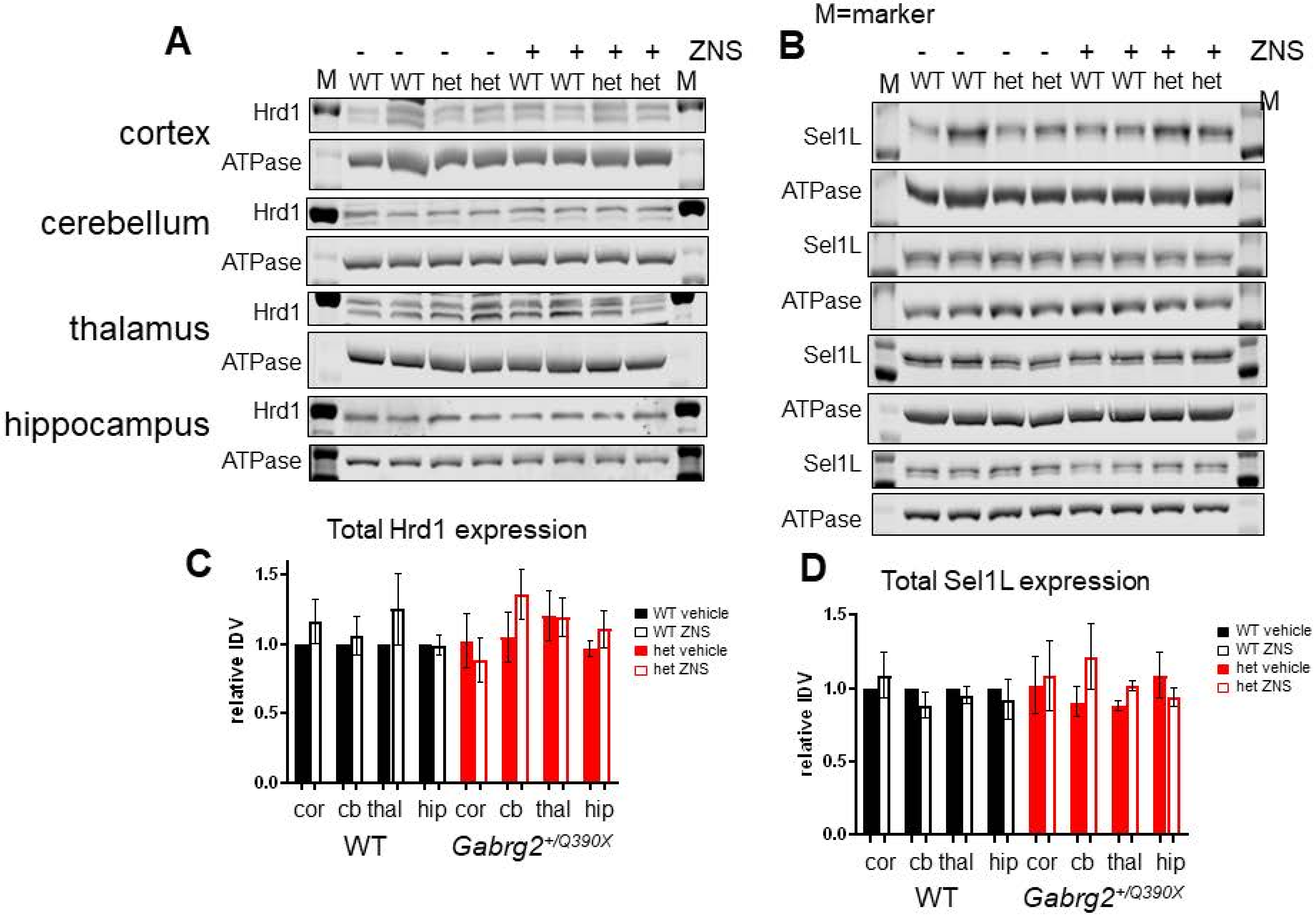

Supplement: Supplementary file 1 [file ijms-25-04601-s001.zip › ijms-2924930-supplementary.pdf]
